# Genetic analysis reveals long-standing population differentiation and high diversity in the rust pathogen *Melampsora lini*

**DOI:** 10.1371/journal.ppat.1008731

**Published:** 2020-08-18

**Authors:** Hanna Susi, Jeremy J. Burdon, Peter H. Thrall, Adnane Nemri, Luke G. Barrett

**Affiliations:** CSIRO Agriculture & Food, Canberra, Australia; University of Neuchatel, SWITZERLAND

## Abstract

A priority for research on infectious disease is to understand how epidemiological and evolutionary processes interact to influence pathogen population dynamics and disease outcomes. However, little is understood about how population adaptation changes across time, how sexual vs. asexual reproduction contribute to the spread of pathogens in wild populations and how diversity measured with neutral and selectively important markers correlates across years. Here, we report results from a long-term study of epidemiological and genetic dynamics within several natural populations of the *Linum marginale-Melampsora lini* plant-pathogen interaction. Using pathogen isolates collected from three populations of wild flax (*L*. *marginale*) spanning 16 annual epidemics, we probe links between pathogen population dynamics, phenotypic variation for infectivity and genomic polymorphism. Pathogen genotyping was performed using 1567 genome-wide SNP loci and sequence data from two infectivity loci (*AvrP123*, *AvrP4*). Pathogen isolates were phenotyped for infectivity using a differential set. Patterns of epidemic development were assessed by conducting surveys of infection prevalence in one population (Kiandra) annually. Bayesian clustering analyses revealed host population and ecotype as key predictors of pathogen genetic structure. Despite strong fluctuations in pathogen population size and severe annual bottlenecks, analysis of molecular variance revealed that pathogen population differentiation was relatively stable over time. Annually, varying levels of clonal spread (0–44.8%) contributed to epidemics. However, within populations, temporal genetic composition was dynamic with rapid turnover of pathogen genotypes, despite the dominance of only four infectivity phenotypes across the entire study period. Furthermore, in the presence of strong fluctuations in population size and migration, spatial selection may maintain pathogen populations that, despite being phenotypically stable, are genetically highly dynamic.

## Author summary

*Melampsora lini* is a rust fungus that infects native flax, *Linum marginale* in south-eastern Australia where its epidemiology and evolution have been intensively studied since 1987. Over that time, substantial diversity in the pathotypic structure of *M*. *lini* has been demonstrated but an understanding of how genetic diversity in pathogen populations is maintained through space and time is lacking. Here we integrated phenotypic, genotypic and epidemiological datasets spanning 16 annual epidemics across three host populations to examine long-term pathogen genetic dynamics. The results show that host ecotype is the dominant selective force in the face of strong bottlenecks and annual patterns of genetic turnover. Results from previous studies indicate that in this geographic region, *M*. *lini* lacks the capacity to reproduce sexually–we thus expected to find limited genetic diversity and evidence for strong clonality influencing genetic dynamics within growing seasons. However, the breadth of genomic coverage provided by the SNP markers revealed high levels of genotypic variation within *M*. *lini* populations. This discovery contrasts with observed phenotypic dynamics as the epidemics of this pathogen were largely dominated by four pathotypes across the study period. Based on a detailed assessment and comparison of pathotypic and genotypic patterns, our study increases the understanding of how genetic diversity is generated and maintained through space and time within wild pathogen populations. The implications for the management of resistance to pathogens in agricultural or conservation contexts are significant: the appearance of clonality may be hiding high levels of pathogen diversity and recombination. Understanding how this diversity is generated could provide new and unique ways to mitigate or suppress the emergence of infectious strains, allowing to efficiently combat harmful diseases.

## Introduction

Evolution within pathogen populations poses ongoing risks for human health, agricultural productivity, and ecosystem structure and function. The response of pathogen populations to novel resistance genes and changing environmental conditions contribute to evolutionary dynamics that drive the emergence and spread of new infective pathogen strains [1–4]. Evolutionary trajectories are expected to vary in space and time and in response to differences in epidemic dynamics and selective pressures imposed by both host and environment. While there has been extensive theoretical work [2, 5–7] investigating pathogen evolution over time, most empirical studies have only assessed pathogen populations over a single growing season [8, 9], or more rarely a few growing seasons [10–13], and often attempt to infer evolutionary dynamics via spatial variation in genetic structure [14, 15] or patterns of local adaptation [16]. There is even less data explicitly linking epidemiology and pathogen population genetic dynamics. The forces that drive population dynamics within vs. among epidemics may diverge due to temporal fluctuations in environment, different selection processes between years [17], at different phases of epidemics [11] and during off-season survival [18].

With respect to antagonistic coevolution between a host and pathogen, there are two theoretically well-grounded scenarios: directional selection and balancing/fluctuating selection [19, 20]. Directional selection (also termed the ‘arms race’) is predicted to lead to ongoing genetic change within pathogen populations via selective sweeps of novel alleles associated with pathogenicity [21–23]. Empirically these dynamics have been tracked by investigating pathogen infectivity [24–26] and host resistance [27] traits in associated populations. The molecular signature of directional evolution has been demonstrated in viruses [26, 28]. The alternative scenario which can maintain polymorphism in populations is balancing or fluctuating selection, where allele frequencies oscillate as a result of negative frequency-dependent selection [19, 20]. This process has been demonstrated experimentally [24, 26, 29]. A combination of the two scenarios may also exist, whereby long periods of balancing selection are interspersed with short periods of directional selection following the emergence of new alleles conferring strong adaptive advantage. To resolve the contribution of each of the two competing dynamics, especially for long lived hosts, more long-term studies investigating the genetic basis of patterns of selection in pathogens are needed.

In addition to genetic change in response to selection imposed by the host, pathogen life-history traits can be important determinants of the frequency and predictability of interactions, influencing pathogen demography and hence patterns of genetic variation [1, 30]. For example, depending on the mode of between-season transmission, some plant pathogen populations experience severe demographic bottlenecks during the off-season [1]. Little is known about how these influence the genetic structure of wild pathogen populations. However, it is assumed that such bottlenecks severely limit the genetic variation that persists within populations from one growing season to the next, following which the remaining genotypes undergo selection where genotypes with high infectivity and reproduction capacity increase in frequency during the subsequent epidemic phase [31]. In addition, dispersal from adjacent populations may result in different combinations of persistent local pathogen isolates and migrant isolates, with their fate being determined by environmental conditions and subsequent selection by hosts [32]. Taken to the extreme, complete pathogen genetic turnover may occur if local populations go extinct during the off-season and host populations are subsequently (re)colonized by strains dispersing from other differentiated populations at the start of the next growing season [33]. Other life-history traits, such as mode of reproduction, may also be critical for determining the genetic structure and diversity of pathogen populations [23]. Thus, for pathogens with clonal or mixed reproduction modes the epidemic phase is generally assumed to be driven by clonal spread, which in turn may strongly influence population genetic structure [31].

Together with pathogen life history traits and spatial structure, varying environmental conditions, host phenotype (e.g. resistance) and life history (e.g. life span, mating system) shape the evolutionary potential of pathogen populations [1, 34] and may lead to adaptation to local conditions [34]. In metapopulations, which typify the situations within which most natural plant pathogens occur, spatial and temporal processes drive the development of both within and among population structures. However, assessing their relative contribution remains challenging. Resolving the spatial scales at which selection by host and environment operates and what genetic structure this creates in natural host-pathogen interactions is of considerable interest with respect to understanding disease evolution [8, 12, 35].

Here we investigate long-term patterns of genetic change in several local populations of the flax rust pathogen *Melampsora lini* and present a detailed analysis of the links between genetic and demographic dynamics in one representative population over the course of 16 annual epidemics through 24 years. Rust epidemics on wild flax, *Linum marginale*, are characterized by strong seasonality and occur within a fragmented structure of partially isolated local populations. This situation results in strongly defined boom and bust dynamics [36] where epidemics that develop during the growing season are typically followed by major declines in pathogen numbers or even local extinction [10, 37]. New infections at the start of a growing season are thought to be initiated from clonal spores (uredia) that over-winter locally on green shoots of host plants, and less occasionally from immigrant spores [37]. Previous studies suggest that epidemics can be dominated by one or few pathotypes [38] but it is unknown how persistent pathotypes are in populations or how pathotypic variation reflects the underlying genotypic variation. To date, molecular evidence elucidating genetic dynamics in natural pathogen populations is rare.

To investigate pathogen evolution, we characterized pathogen variation with a suite of markers–SNPs, infectivity genes and infectivity phenotypes. Characterization of phenotypic infectivity in the *Linum-Melampsora* system is well established using a host differential set–a standard set of plant lines with differing resistance backgrounds. [39, 40]. In addition, several loci important to pathogen infection outcomes have previously been cloned [17, 41], and we examine variation at two of these loci (*AvrP123; AvrP4*) that have relatively high levels of sequence variation and are under strong diversifying selection [41]. We analysed variation in 629 *M*. *lini* isolates collected between the years 1987 and 2010 in three sub-alpine populations. The host populations that carry them are geographically close to each other, but experience different ecological conditions, and are morphologically and genetically differentiated, such that the ‘hill’ (Kiandra) and ‘bog’ (P1 and P2) host populations represent different ecotypes [36]. At a broader geographical scale the Australian population of *M*. *lini* is represented by two distinct lineages that differ in their heterozygosity, an AB lineage being common in the sub-alpine region and an AA lineage in areas with hot summer conditions [42]. We test the following hypotheses: 1. Pathogen populations differ markedly within and among sites, across years and are further structured by host ecotype. We expect selection imposed by the host ecotype to play a major role in shaping pathogen populations; and epidemics to be the driver of temporal genetic structure. 2. Pathogens reproduce and spread asexually, strongly limiting population genetic diversity within growing seasons.

## Results

### Population structure

We investigated whether population differentiation by host ecotype is stable over time in genetic and phenotypic variation for 629 *M*. *lini* isolates collected between 1987 and 2010 from three populations (Table 1). To do this, we used SNP markers, sequence data from infectivity genes (Avr haplotypes) and infectivity phenotypes (pathotypes) to probe variation within and among populations.

**Table 1:** The number of analysed *Melampsora lini* samples in populations Kiandra, P1, and P2 across years 1987–2010. The number of samples (*n*) observed Shannon diversity index (*H*), and richness (*R*) as number of distinct SNP genotyped multi-locus genotypes (MLG), SNP genotyped DAPC clusters (Cluster), infectivity phenotypes analysed with differential set (Pathotype), and Avr gene haplotypes (Avr gene).

	Kiandra												P1												P2												All											
Year	MLG			Cluster			Pathotype			Avr gene			MLG			Cluster			Pathotype			Avr gene			MLG			Cluster			Pathotype			Avr gene			MLG			Cluster			Pathotype			Avr gene		
* *	*n*	*R*	*H*	*n*	*R*	*H*	*n*	*R*	*H*	*n*	*R*	*H*	*n*	*R*	*H*	*n*	*R*	*H*	*n*	*R*	*H*	*n*	*R*	*H*	*n*	*R*	*H*	*n*	*R*	*H*	*n*	*R*	*H*	*n*	*R*	*H*	*n*	*R*	*H*	*n*	*R*	*H*	*n*	*R*	*H*	*n*	*R*	*H*
1987	42	40	3.7	42	4	1.1	38	6	0.8	37	3	0.2	13	13	2.6	13	4	1.1	13	5	1.3	11	2	0.3	13	10	2.1	13	2	0.4	13	7	1.7	9	2	0.6	68	62	4.1	68	5	1.5	64	13	1.6	57	4	0.8
1988	74	68	4.2	74	4	0.9	58	5	0.6	68	3	0.5	22	21	3	22	4	1	17	5	1.2	20	2	0.2	19	14	2.6	19	4	0.6	19	5	1.4	16	1	0	115	102	4.5	115	5	1.4	94	11	1.4	104	4	0.8
1989	33	33	3.5	33	3	1.1	25	7	1.2	30	4	0.8	8	8	2.1	8	2	0.4	8	3	0.7	8	1	0	4	3	1	4	1	0	4	3	1.3	4	1	0	45	39	3.6	45	4	1.2	37	11	1.8	42	4	1.3
1990	27	24	3.1	27	5	1.5	24	5	1.1	23	5	1.3	12	11	2.4	12	4	1	10	6	1.6	11	2	0.3	0	-	-	0	-	-	0	-	-	0	-	-	39	35	3.5	39	5	1.5	34	8	1.5	34	5	1.4
1991	22	20	2.5	22	6	1.5	15	7	1.6	15	4	1.1	12	12	2.5	12	5	1.5	10	3	0.6	10	3	0.7	0	-	-	0	-	-	0	-	-	0	-	-	34	29	3.1	34	6	1.6	25	8	1.2	25	4	1.2
1992	25	25	3.2	25	4	1.3	18	3	0.7	22	4	0.8	18	17	2.8	18	3	0.7	12	3	1	11	1	0	20	16	2.7	20	3	0.7	20	5	1.3	11	2	0.3	63	48	4	63	5	1.4	50	5	1.3	44	4	1
1994	1	1	0	1	1	0	1	1	0	0	-	-	6	5	1.6	6	3	1	4	4	1.3	6	2	0.5	12	10	2.2	12	2	0.5	12	2	0.7	11	1	0	19	15	2.6	19	4	1.1	17	7	1.7	17	2	0.6
1995	2	2	0.7	2	2	0	0	-	-	0	-	-	3	3	1.1	3	2	0.6	3	3	1.1	3	3	1.1	6	6	1.8	6	2	0.5	6	2	0.5	6	1	0	11	11	2.4	11	4	1.2	9	4	1.1	9	3	0.7
1996	57	40	3.5	57	4	0.7	47	6	0.9	49	2	0.1	6	6	1.8	6	2	0.5	1	1	0	4	3	0	4	3	1	4	1	0	4	2	0.6	4	1	0	67	48	3.7	67	5	1	52	7	1.2	57	3	0.5
1997	31	26	3.2	31	4	0.8	30	8	1.2	26	3	0.6	28	19	2.7	28	4	0.8	10	8	1.9	25	3	0.6	0	-	-	0	-	-	0	-	-	0	-	-	59	45	3.7	59	5	1.3	40	16	1.7	51	4	1.1
2002	27	24	3.1	27	5	1.3	9	4	1	0	-	-	0	-	-	0	-	-	0	-	-	0	-	-	0	-	0	-	-	0	-	-	0	-	-	-	27	24	3.1	27	5	1.3	9	4	0.6	0	2	0.4
2004	31	29	3.4	31	3	0.4	7	3	1	30	1	0	0	-	-	0	-	-	0	-	-	0	-	-	0	-	-	0	-	-	0	-	-	0	-	-	31	29	3.3	31	2	0.4	7	3	1	30	1	0
2005	18	18	2.9	18	3	0.2	0	0	-	17	2	0.2	0	-	-	0	-	-	0	-	-	0	-	-	0	-	-	0	-	-	0	-	-	0	-	-	18	18	2.9	18	2	0.2	0	0	-	17	2	0.2
2006	12	12	2.5	12	3	0.6	8	5	1.4	17	2	0.7	0	-	-	0	-	-	0	-	-	0	-	-	0	-	-	0	-	-	0	-	-	0	-	-	12	12	2.5	12	2	0.6	8	5	0.7	17	2	0.3
2008	11	10	2.3	11	4	1	5	5	1.6	11	2	0.8	0	-	-	0	-	-	0	-	-	0	-	-	0	-	-	0	-	-	0	-	-	0	-	-	11	10	2.3	11	3	1	5	5	1	11	3	0.9
2010	0	-	-	0	-	-	0	-	-	11	3	0.3	10	9	2.2	10	3	0.6	0	-	-	9	1	0	0	-	-	0	-	-	0	-	-	0	-	-	10	10	2.2	10	3	0.6	0	-	-	20	1	0
Total	413	292	5.6	413	6	1.3	285	41	1.3	356	5	0.7	138	104	4.5	138	6	1.3	88	22	2	118	4	0.6	78	46	3.4	78	4	0.6	78	8	1.7	61	3	0.2	629	416	5.7	629	6	1.6	451	56	1.9	535	5	1

**Fig 1:**
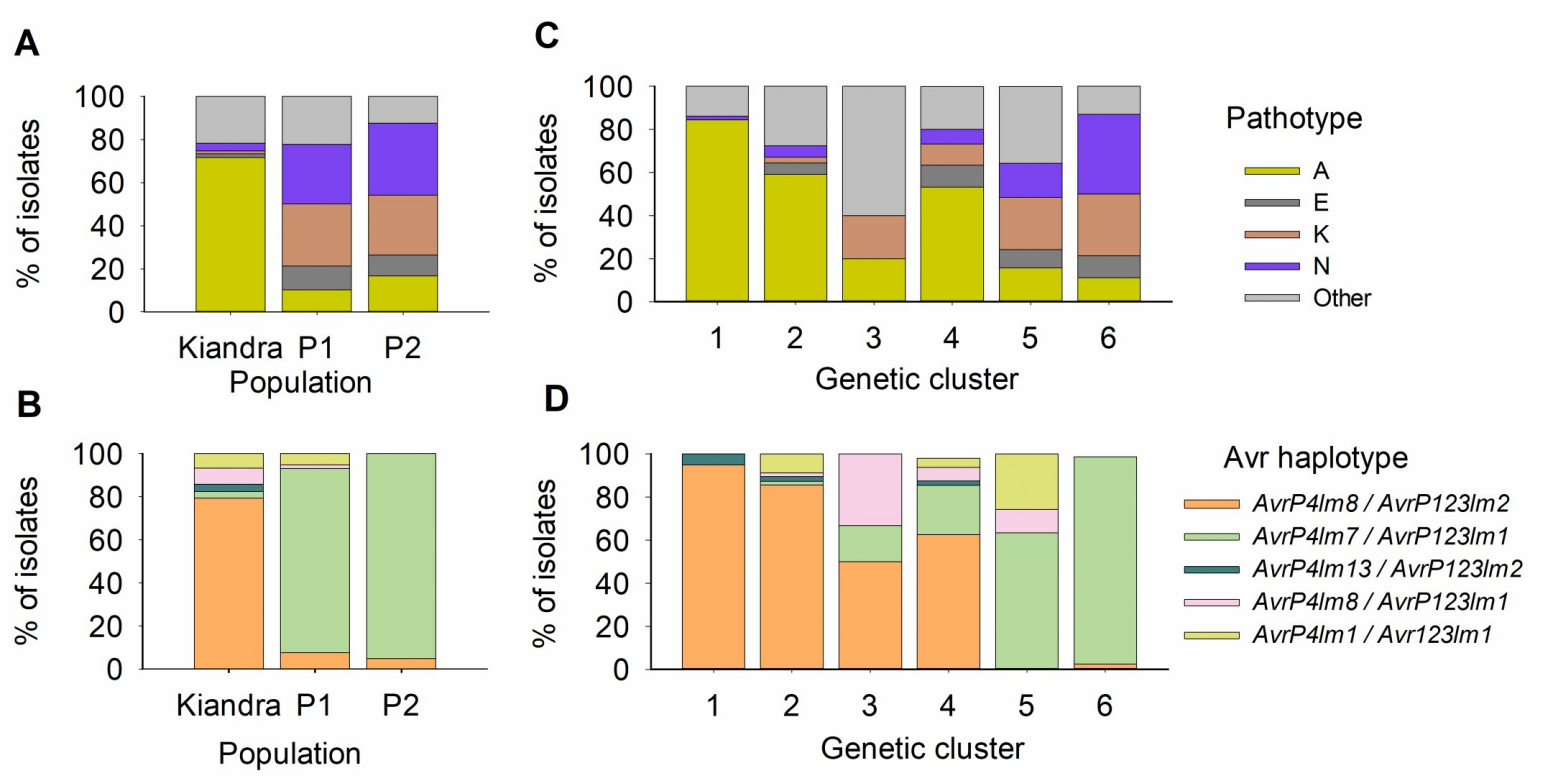
*Melampsora lini* populations and genetic clusters differ in their Avr genotype and pathotype compositions. The distribution of *Melampsora lini* isolates by study site: (A) pathotypes; (B) Avr genotypes; and by genetic clusters (C) pathotypes; (D) Avr genotypes.

**Fig 2:**
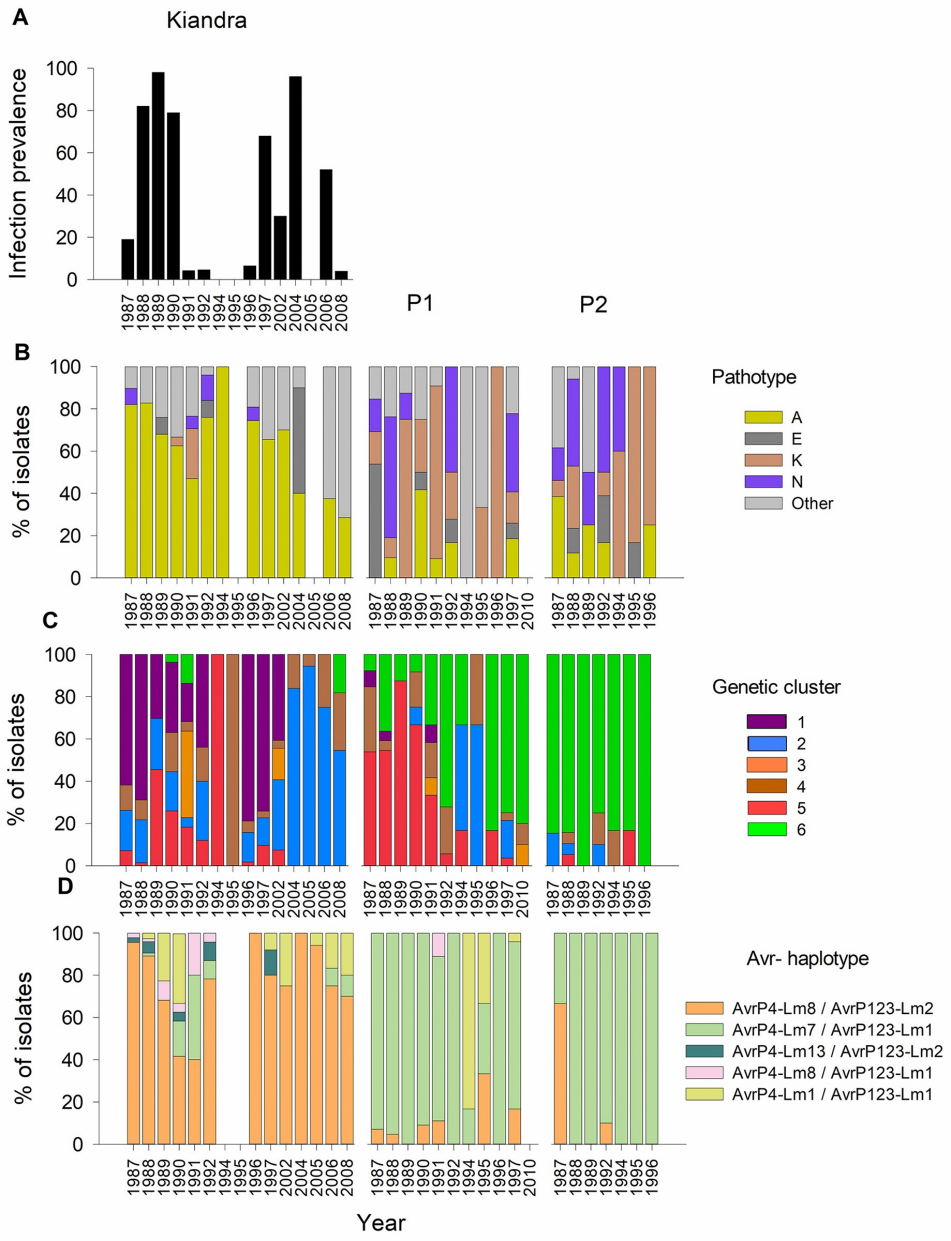
*Melampsora lini* population differentiation and temporal change revealed by genetic clusters, Avr genotypes and pathotypes. **(**A) Annual infection prevalence of *Melampsora lini* on *Linum marginale* in Kiandra over years 1987–2008. The distribution of (B) pathotypes, (C) genetic clusters, and (D) Avr genotypes of *Melampsora lini* populations Kiandra, P1 and P2 over years 1987–2010.

Analysis of similarities (ANOSIM)[43] of infectivity phenotypes indicated populations were moderately differentiated (*R* = 0.36; *P* = 0.001; Table 2; Figs 1C and 2A), while differentiation by ecotype was strong (*R* = 0.50; *P* = 0.001; Table 2). ANOSIM indicated no differentiation between years. Phenotypic data from inoculation trials using the *L*. *marginale* differential set revealed the presence of 56 different pathotypes over the entire period of the study (1987–2010). Pathotypic diversity was highest in P1 (Shannon *H* = 2.02) vs Kiandra or P2 (*H* = 1.28 and 1.74 respectively; Table 1). Pathogen populations were dominated (82.0% of the phenotyped isolates) by four common pathotypes (designated A (50.3%), E (5.3%), K (12.1%) and N (14.1%)), with other pathotypes detected periodically (Figs 1A and 2B). Pathotype A was by far the most common in the Kiandra population whereas A, E, K and N were the major pathotypes in P1 and P2 (Fig 1A).

**Table 2:** Results from analysis of similarities (ANOSIM) obtained using genome wide SNP genotyping clusters (Cluster), infectivity phenotypes (Pathotypes), and infectivity gene genotypes (Avr gene) for isolates of *Melampsora lini* originating from three populations (Kiandra, P1 and P2) over the years 1987–2008.

	Ecotype		Population		Year	
Marker	*R*	*P*	*R*	*P*	*R*	*P*
Cluster	0.59	0.001	0.45	0.001	0.10	0.808
Pathotype	0.50	0.001	0.36	0.001	0.03	0.625
Avr gene	0.92	0.001	0.66	0.001	0.08	0.733

We SNP-genotyped *M*. *lini* isolates from all three populations using Nextrad sequencing and performed hierarchical AMOVA to assess how SNP variation was distributed among populations, years, and pathotypes (Table 3). The analysis revealed significant differences in population structure among the three populations (8.6%) as well as among years within populations (9.7%; Table 3). A major proportion of the variation (81.7%) was found within years (Table 3). When AMOVA was performed including pathotypes as a hierarchical level in the model, small significant differences were found among populations (0.5%), while pathotypes within populations explained 25.2% of the variation (Table 3). Variation between years within pathotype and population was non-significant (2.0%; *P* = 0.07) and variation within year again explained the largest proportion of the variation (72.3%; Table 2). To assess the contribution of clonality to changes in composition over time, analyses were also performed using clone corrected data; these demonstrated the same distribution of variation although the between years / within pathotypes interaction term lost statistical significance (S1 Table).

**Table 3:** Results of hierarchical analysis of molecular variance (AMOVA) using genome wide SNP genotyping, and infectivity gene (AvrP123 and AvrP4) genotyping for isolates of *Melampsora lini* originating from three populations (Kiandra, P1 and P2) over the years 1987–2008. Results are presented for two analyses of SNP genotypes; for the whole dataset (N = 629) population and year as hierarchical levels, and the dataset of *Melampsora lini* isolates that were pathotyped (N = 451) containing pathotypes as a third hierarchical level. Results for Avr pathotypes are presented for those for which infectivity genes were characterised (N = 539) with population and year as hierarchical levels.

** SNP genotyping**	***Df***	***Sum of Squares***	***Mean Squares***	***% Variance***	***P***
**Between populations**	2	0.249	0.124	8.6	0.001
**Between years within populations**	30	0.598	0.019	9.7	0.001
**Within populations within year**	596	3.7	0.006	81.7	0.001
**Total**	628	4.547	0.007	100	
					
**Between populations**	2	0.17	0.085	0.5	0.01
**Between pathotypes within populations**	73	0.87	0.012	25.2	0.001
**WIthin populations and pathotypes between years**	62	0.34	0.006	2	0.07
**Within populations and pathotypes within year**	353	1.7	0.005	72.3	0.001
**Total**	490	3.09	0.006	100	
**Avr gene genotyping**	***Df***	***Sum of Squares***	***Mean Squares***	***% Variance***	***P***
**Between populations**	2	146.5	0.56	66.8	0.001
**Between years within populations**	27	25.8	0.05	5.5	0.001
**Within populations within year**	453	106.3	0.23	27.7	0.001
**Total**	482	278.6	0.84	100	

**Fig 3:**
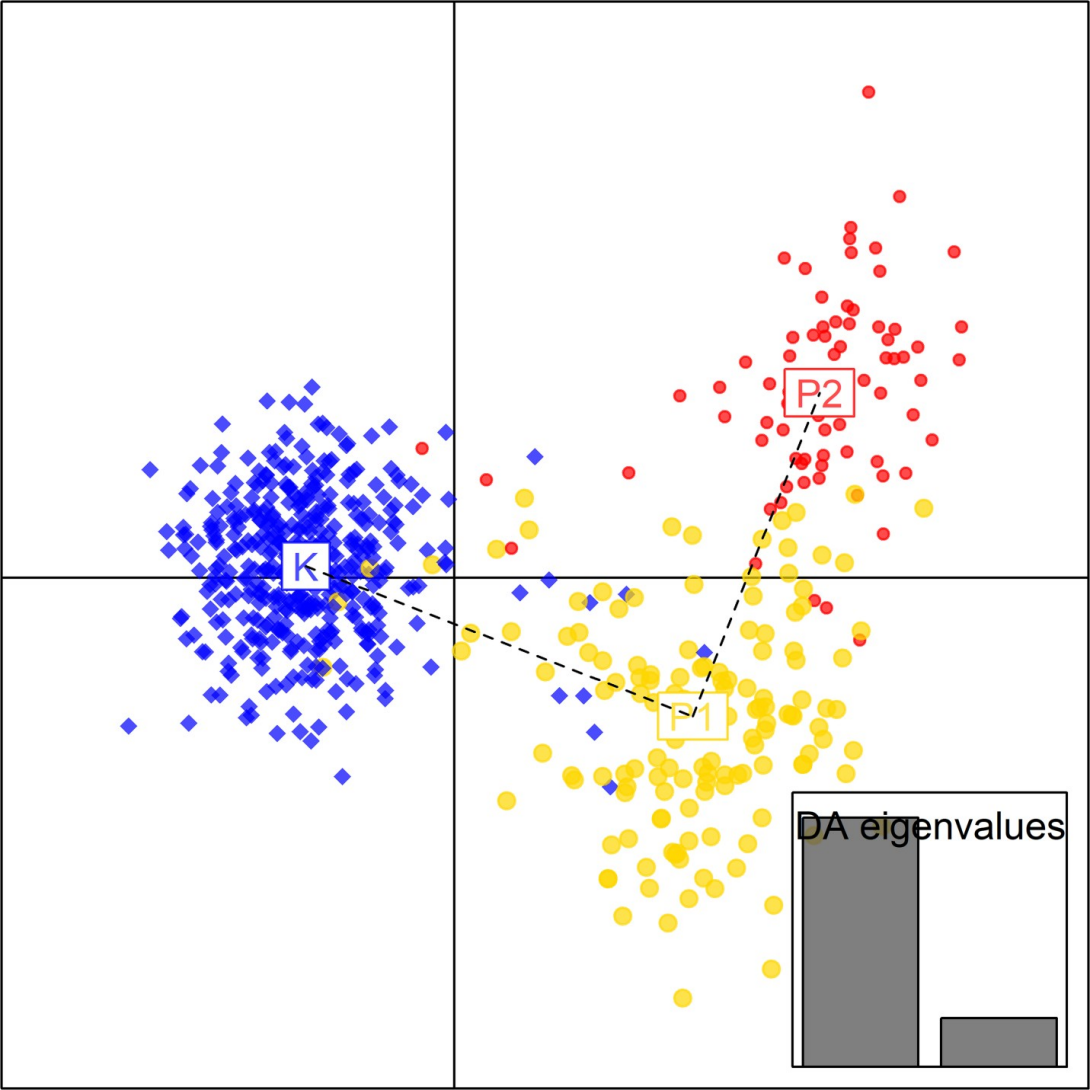
Principal component analysis on SNP genotyping of *Melampsora lini* shows population differentiation. Distribution of the genetic variation of *Melampsora lini* isolates collected from populations Kiandra (blue), P1 (yellow) and P2 (red) based on principal component analysis.

Following this, we performed principal component analysis (PCA) of the SNP data based on Prevosti’s distances. The PCA revealed clear differentiation among populations (Fig 3). The first principal component clearly separated P1 and P2 from Kiandra (Fig 3). P2 was differentiated from P1 and Kiandra on the second principal component (Fig 3).

**Fig 4:**
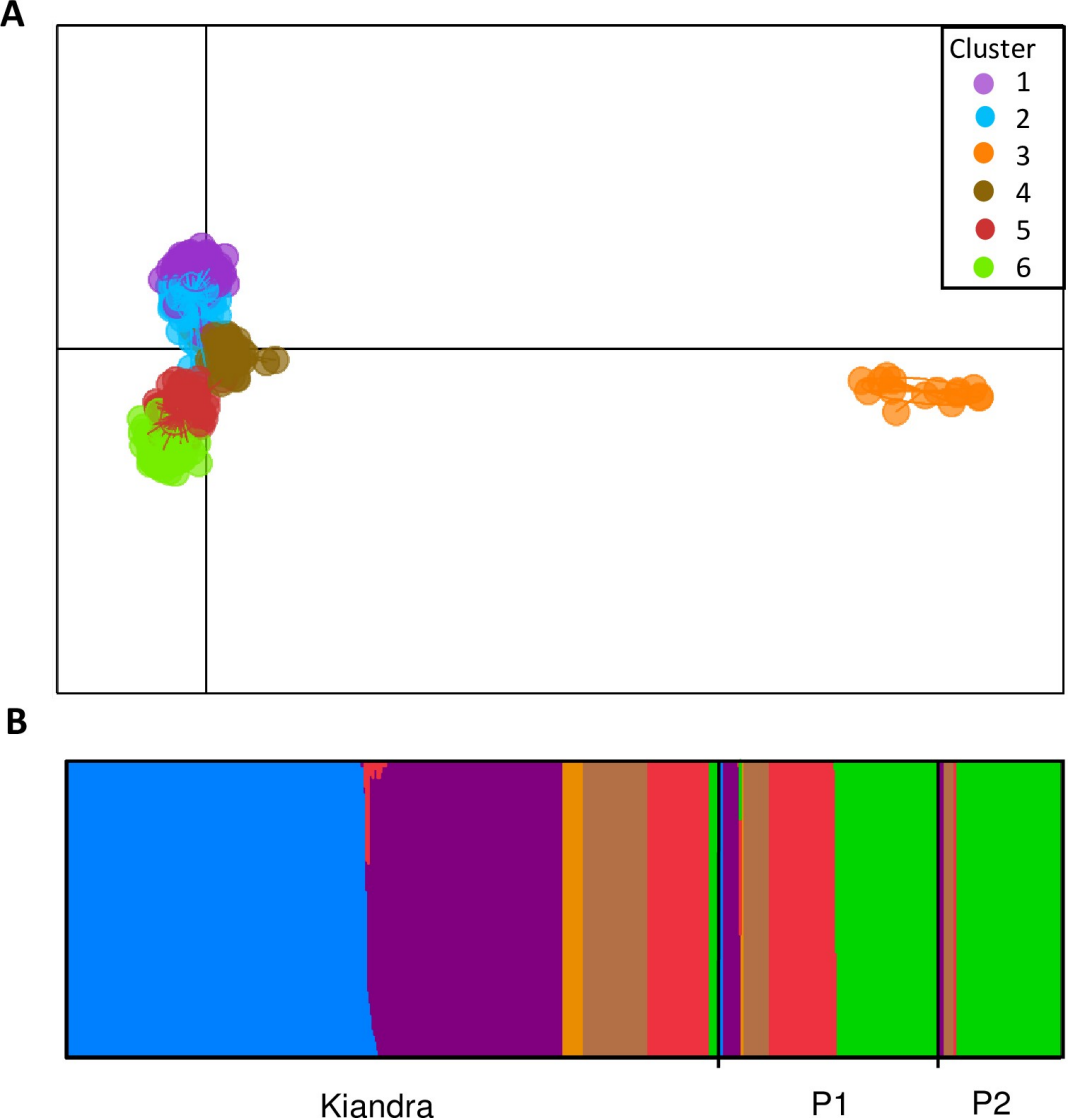
DAPC clustering of *Melampsora lini* isolates reveals six clusters with low levels of hybridization between clusters. (A) Genetic clusters of *Melampsora lini* obtained by DAPC. (B) Distribution of the clusters in the populations and their assignment probability, each bar represents percentage probability to genetic group(s).

Discriminant analysis of principal components (DAPC) revealed six clusters with assignment probability of 99.2%, including one cluster (Cluster 3) that was strongly differentiated from all the others along the first principal component axis and had a distant placement in a neighbour-joining tree (Fig 4A; S1 Fig). The number of samples with intermediate clusters was relatively low; only a minor fraction of the samples (1.1%) had less than 70% assignment probability. In ANOSIM analysis based on the DAPC assignments, the three populations differed with some overlap in genetic composition (*R* = 0.45; *P* = 0.001; Table 2; Figs 2C and 4B), while the differentiation by ecotype was stronger (*R* = 0.59; *P* = 0.001; Table 2). In ANOSIM, years were not significantly differentiated. Kiandra was the most diverse population when the Shannon diversity of genetic clusters was analysed (*H* = 1.34; vs in P1 *H* = 1.31 and P2 *H* = 0.62). With regards to infectivity phenotypes, the clusters consisted of several different pathotypes (Fig 1C).

In addition to SNP markers, we also characterized nucleotide variation at loci underlying pathogen infectivity. Specifically, we PCR amplified and sequenced alleles of the infectivity genes *AvrP4* and *AvrP123* (as per [41]). We constructed multi-locus *AvrP4*/*AvrP123* haplotypes by combining the sequenced infectivity genes for each isolate and found five commonly occurring haplotypes (Fig 1B), comprising 92.5% of the samples. *AvrP4/AvrP123* haplotypes were variable among populations and genetic clusters (Fig 1B and 1D). Analysis of similarities (ANOSIM) on *AvrP4*/*AvrP123* haplotypes revealed differentiation by population (*R* = 0.66; *P* = 0.001; Table 2) and strong differentiation by ecotype (*R* = 0.92; *P* = 0.001; Table 2). Variation by year was weaker (*R* = 0.08; *P* = 0.733; Table 2). Hierarchical analysis of molecular variation (AMOVA) for the *AvrP4* and *AvrP123* genes revealed strong differentiation among populations, explaining 66.8% of the variation (Table 3). In contrast, variation among years within each population accounted for only 5.5% of the variation (Table 3). With respect to haplotypic diversity, we found that Kiandra was the most diverse population (Shannon (*H)* = 0.70, vs P1 *H* = 0.55, and P2 *H* = 0.20; Table 1; Figs 1B and 2D).

### Mixed reproduction

To understand whether clonal increase drives annual epidemics, we first assigned isolates to multi-locus genotypes (MLGs). To avoid potential underestimation of clonality due to sequencing error, we used a 5% error rate (the maximum error rate observed from the replicate sequencing of three control isolates). After filtering the SNP data using this 5% error rate, the data indicated most isolates were genetically unique– 83.9% of the MLGs were sampled only once. Consequently, a relatively high number of MLGs were found in each of the sampled populations (Kiandra: 292 MLGs out of 413 isolates; P1: 103 out of 138 isolates; P2: 43 out of 78 isolates). The 67 MLGs sampled more than once constituted 44.1% of the sampled isolates. When we analysed the prevalence of non-unique MLGs we found that each appeared between 2–13 times (mean 4.05) during the study period. Within annual epidemics, clonal spread comprised 0–44.8% of the isolates collected per season (Table 4).

**Table 4:** Assessment of clonal vs potentially sexual reproduction in *Melampsora lini* across seven overwintering seasons. Results from the *Cloncase* analysis performed using the whole annual pathogen sample comprising isolates from the Kiandra, P1 and P2 populations was used to assess effective population size (*Ne*) and potentially sexual reproduction (*s*). The highest number of clonemates shows the number of isolates belonging to the most abundant MLG. Asterisks indicate years when analysis was not done due to lack of samples or small sample size.

Year	*n*	MLGs	Clone mates	Highest number of clone mates	% clonal isolates	Effective population size, *Ne*	Sexual reproduction rate, *s*
1987*	68	62	3	4	11.94	-	-
**1988**	115	102	11	3	20.86	10580	0.4%
**1989**	45	39	3	3	17.80	3496	0.06%
**1990**	39	35	2	3	18.42	1672	83%
**1991**	34	29	3	5	32.35	481	78%
**1992**	63	55	4	3	15.00	2658	98%
1995*	11	11	0	0	0.0	-	-
**1996**	67	48	11	5	44.77	686	78%
**1997**	59	45	7	5	34.50	3016	21%

To estimate effective population sizes and reproduction mode during the winter season, we used MLG occurrences across two consecutive years in a *Cloncase* analysis [44]. *Cloncase* is a program that implements algorithms to estimate the expected sexual and clonal reproduction using MLG frequencies and the estimates of clonal cycles before and after the winter season. However, it does not use allele information obtained by SNP genotyping, thus does not produce any estimate of linkage between loci or similarity of MLGs. The analysis estimated variable rates of potentially sexual reproduction across years (0.06–98% of the population), and high effective population sizes from 481 to 10580 (Table 4). We then conducted rarefaction analysis to estimate the sampling size needed to capture all MLGs present in a population. The rarefaction curve further indicated that not all genotypic variation present in the three populations was sampled (S2 Fig).

We used the SNP genotype data to test for signs of recombination. *Phi* tests [45] applied separately to each cluster indicated that genetic recombination is highly likely to have occurred within each of the clusters, although not in every cluster within a population (S2 Table, S3 Table). In Kiandra, significant evidence for recombination was detected in only two of the clusters whereas in P1 and P2 recombination was more frequent (S2 Table, S3 Table). Likewise, significant evidence for recombination was detected within each of the populations, although not in every year (S2 Table, S3 Table). Significant levels of linkage disequilibrium (LD) were found in 13 out of 16 years evaluated; whereas no significant LD was observed for the remaining 3 years (1992, 1997 and 2008; S4 Table). Despite evidence for recombination, significant LD was observed within all clusters and populations (S2 Table). Together, these results indicate that some form of genetic exchange among isolates does occur, most likely within clusters, but that genetic exchange is limited and mating is far from random.

### Populations change through time

We tested whether the information obtained from neutral and selectively important genetic and phenotypic markers revealed similar patterns of pathogen population evolution through time. To do this, we used both genetic and phenotypic data, including information collected from the long-term epidemiological surveys of the Kiandra site, where infection prevalence varied markedly among years, ranging from 0 to 98% (Fig 2A). While infection prevalence also varied seasonally at P1 and P2 (JJ Burdon pers. obs.), epidemiological data was not collected at those sites. Comparison of genetic and phenotypic patterns with the epidemiological data from Kiandra revealed dynamic among–year fluctuations in population genetic structure consistent with observed boom and bust disease dynamics and pathogen population bottlenecks (Fig 2A). For example, in 1996 (a low epidemic year; Fig 2A) the previously common Cluster 1 isolates were found in Kiandra after two low epidemic years when previously rare clusters 3 and 4 were sampled (Fig 2C). In 2004 (a high epidemic year), Cluster 2 isolates replaced the previously common Cluster 1. However, in contrast to 1996, this resulted in a long-term replacement such that Cluster 2 isolates were maintained at high frequencies in subsequent years, while Cluster 1 isolates were not sampled again (Fig 2C). In other years, uncommon clusters were present in various combinations, but fluctuated in relative frequency (Fig 2C). Likewise, the genetic composition of populations P1 and P2 also showed dynamic changes among years. In P1, isolates assigned to Cluster 6 gradually replaced isolates assigned to Cluster 5 (Fig 2C). The neighbour-joining tree (S1 Fig) indicated some overlap between clusters 2, 4, and 6, suggesting that the clusters increasing in frequency (2 and 6) may have evolved from cluster 4. In both Kiandra and P1, isolates assigned to Cluster 3 were sampled for the first time in 1991 and again in 2002, and 2010 (placement of Cluster 3 in a neighbour-joining tree shown in S1 Fig). Heterozygosity within Cluster 3 was significantly lower (56.8 ± 1.31%) than in the other clusters (Cluster 1 = 74.8%; 2 = 73.2%; 4 = 73.5; 5 = 71.1%; 6 = 74.4%). This suggests that Cluster 3 may represent the largely homozygous AA lineage of *M*. *lini* that dominates in the distant plains region [10]; its simultaneous appearance in both Kiandra and P1 in 1991 likely indicates a migration event.

Pathotype and *AvrP4/AvrP123* haplotype dynamics in the three host populations showed similar changes to genetic cluster dynamics through time (Mantel test Avr haplotypes vs genetic clusters *r*_*M*_ = 0.91; pathotypes vs genetic clusters *r*_*M*_ = 0.91; and Avr haplotypes vs pathotypes *r*_*M*_ = 0.87; Table 5; Fig 2B–2D). Pathotype A (dominant at Kiandra for the first 15 years) showed a decline in frequency over time that may indicate the results of selection. In contrast, pathotype E, which was initially sampled at very low frequencies, seemingly going locally extinct for periods of time, strongly increased in frequency in 2004 following reintroduction from the wider metapopulation. This is interesting, due to the concordance with the replacement of Cluster 1 isolates by Cluster 2 (Fig 2B and 2C) it may indicate change in the selection in the population. In populations P1 and P2 four common pathotypes, K, N, A and E, were sampled with variable annual frequencies (Fig 2B). In addition to the four common pathotypes, all populations included new pathotypes in each of the sampled years, many of which (63.7%) were only sampled in one year. Similarly, *AvrP4/AvrP123* haplotype frequencies were dynamic through time (Fig 2D). In Kiandra, *AvrP4*/*AvrP123* haplotype diversity decreased over time, with only three of the five common haplotypes present after 2002 (Fig 2D).

**Table 5:** Correlation of *Melampsora lini* population diversity measured with different markers and infection prevalence. Diversity of SNP genotyped genetic clusters (Cluster), infection phenotypes measures with the host differential set (Pathotype) and Avr gene haplotypes (Avr gene) and infection prevalence analysed with Mantel tests.

		Kiandra		P1		P2		All	
		*r*_*M*_	*P*	*r*_*M*_	*P*	*r*_*M*_	*P*	*r*_*M*_	*P*
Cluster	Pathotype	0.89	0.001	0.06	0.335	0.36	0.055	0.91	0.001
Cluster	Avr gene	0.62	0.001	0.27	0.074	0.05	0.305	0.91	0.001
Avr gene	Pathotype	0.53	0.001	0.08	0.364	0.57	0.001	0.87	0.001
Cluster	Infection	0.47	0.01	-	-	-	-	-	-
Avr gene	Infection	0.65	0.001	-	-	-	-	-	-
Pathotype	Infection	0.48	0.005	-	-	-	-	-	-

**Fig 5:**
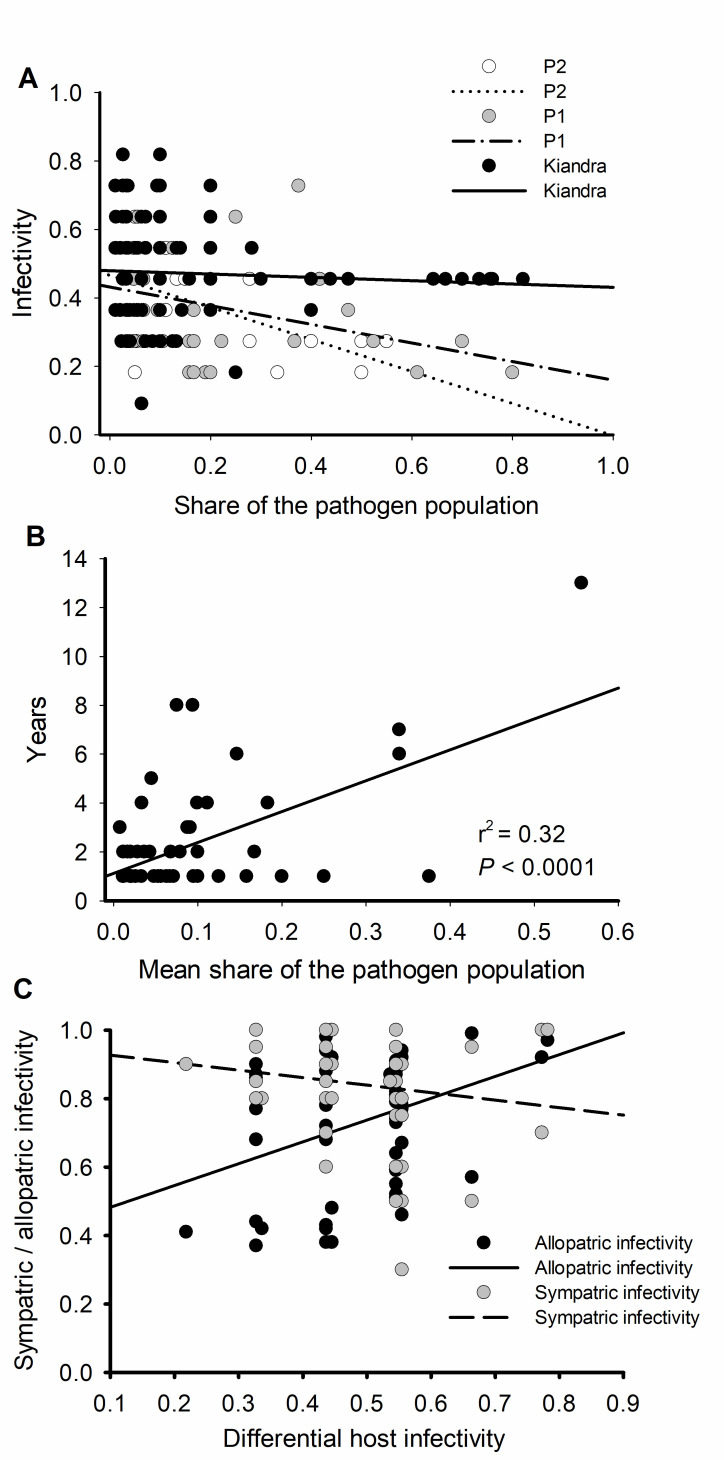
Linking *Melampsora lini* pathotype infectivity and share of the population. **(**A) Correlation between *Melampsora lini* pathotypes share of the population in three populations, Kiandra, P1 and P2, and infectivity over the *Linum marginale* differential host set. (B) Correlation between the pathotype’s mean share of the population and years of prevalence in the population. (C) Correlation between the differential set measured pathogen infectivity and infectivity measured using sympatric hosts (grey circles, dashed line) and allopatric hosts (black circles, solid line).

To investigate processes influencing variation in infectivity among populations, we explored the relationship between pathotype infectivity (as assessed on the *L*. *marginale* differential set) and pathotype prevalence in the study populations. The expectation is that pathotypes with greater host range will be more frequent than their counterparts with a narrower host range in highly resistant populations whereas in susceptible populations the opposite is expected. Our results showed that infectivity was negatively correlated with prevalence (d.f. = 1,45; F = 13.99; P = 0.0003). While there was no significant effect of pathogen population (d.f. = 2,145; F = 1.24; P = 0.292; Fig 5A), there was a significant interaction between population and prevalence (d.f. = 2,45; F = 3.77; P = 0.0254; Fig 5A). In other words, the relationship between infectivity and prevalence varied depending on the population from which pathogens were sampled. To investigate drivers of pathotype dynamics through time we examined the relationship between pathotype prevalence and persistence (i.e. the number of years in which a given pathotype was present in the population). We found that pathogen prevalence and persistence were positively correlated (d.f. = 1,72; F = 51.2; P < 0.0001; Fig 5B), but there was no significant effect of a pathotype’s proportion of the population (d.f. = 2,72; F = 0.48; P = 0.6203).

The differential set of *L*. *marginale* genotypes includes host lines from across Australia. To link it to a local context, we tested whether measures of infectivity on differentials correlated with infectivity measured with hosts originating from the same population, or with hosts originating from other populations within the same geographical area. Thus, for a subset of isolates for which data were available, we analysed correlations between infectivity measured using the differential set and infectivity measured on a set of local sympatric (from the same population) and allopatric (from a more distant population) hosts used in a different study [17]. While infectivity on the differential set did not correlate with that measured on sympatric hosts (R^2^ = 0.02; d.f. = 1,55; F = 0.01; P = 0.9106; Fig 5C), there was a positive correlation with infectivity measured on allopatric hosts (R2 = 0.14; d.f. = 1,58; F = 9.29; P = 0.0035; Fig 5C).

## Materials and methods

### The host-pathogen system

The *Linum marginale*—*Melampsora lini* association has been extensively described [17, 36–38] but essentially consists of an annual interaction between an herbaceous, perennial host and a host-specific rust fungus, in which harsh winter conditions precipitate major pathogen population crashes. The pathogen spreads through up to 16 asexual cycles during the growing season (November to April) and overwinters in remaining plant parts clonally as mycelium, or as teliospores going through the sexual cycle [10]. The potential number of clonal cycles in a season was estimated on the basis of knowledge built up through many years of infectivity testing in the laboratory coupled with field observations and estimates of the average season length based on temperature patterns through the summer growing period and typical occurrence of first frost days in autumn. Previous assessment of molecular markers, pathogen infectivity genes and pathotype variation within the study area [10, 42, 46] found strong patterns of LD consistent with limited sexual recombination.

### The study sites

The host and pathogen populations surveyed and sampled for this study are located within the Kiandra Plain, in the northern part of Kosciuzko National Park, New South Wales, Australia. The main site (Kiandra) is located on a hillside; at this site epidemic development was assessed in addition to the collection of individual pathogen samples. At the other two sites (P1, P2), located on flat areas adjacent to stream courses, only pathogen samples were collected. The host plant occurs in these sites as two ecotypes: hill (Kiandra), and bog (P1 and P2) which differ both morphologically (bog plants are shorter with flowers little bigger than surrounding sepals, resulting in flowers often not opening completely) and genetically (isozyme patterns) [36]. Population P1 is approximately 350 metres from the Kiandra site, while P2 is approximately 2.5 km away.

### Collection, purification and pathotype analysis of isolates

The number of sampling times changed across the course of the study; in the early years samples were collected at both the start of epidemics (just a few samples) as well as at epidemic peaks (many samples). In later years most sampling was done during the epidemic peak. The sampling intensity was scaled to the epidemic size. Thus, in years in which conditions were unfavourable to the pathogen (i.e. no epidemic occurred), the number of samples collected was small (e.g. Kiandra in the years 1994–1995). On each sampling occasion in each year, pathogen sampling was conducted by examining plants chosen haphazardly across the entire site; this was done deliberately to avoid sampling the same local infection focus multiple times. No attempt was made to try to revisit the same host individuals across years. The infectivity of individual isolates from the three sites was assessed over multiple years (Kiandra, 285 samples: 1987–1997, 2002, 2004–06, 2008; P1, 88 samples: 1987–1997; P2, 78 samples: 1987–1989, 1992–1997; Table 1). Altogether 451 samples were used for pathotyping. Single pustules were sampled from unique plants in the field. Pure strains were subsequently produced by starting propagation on a universally susceptible host from single pustules as the urediospores within a pustule are clonal. Details on collection, purification and storage of isolates are available elsewhere [36–38].

The infectivity pathotype of each *M*. *lini* isolate was assessed on 12 differentially resistant lines of *L*. *marginale* previously identified as carrying different resistance genes or alleles [47]. Infectivity was assessed by inoculating young shoots of each of the 12 differential lines and 12–14 days later assessing infection type reactions (37). Any tests giving ambiguous results were repeated. Pathotype designations therefore represent a unique combination of resistant and susceptible responses of the differential lines to individual pathogen isolates.

To test whether infectivity measured on differential hosts correlates with infectivity on sympatric and allopatric (i.e. distant) hosts, inoculations were performed on hosts and pathogens collected from six other populations, G1, G3, SH1, SH2, WHP1 and WHP2 by inoculating the pathogens on hosts from their own population and on hosts from their five allopatric populations as described elsewhere [17].

### DNA extraction and genotyping

For each isolate, total genomic DNA was extracted from approximately 100 mg of urediospores using a DNeasy plant mini kit (Qiagen, Germany) and quantified using a Qubit 3.0 fluorometer. In addition, we genotyped three samples as duplicates in separate plates to estimate sequencing errors. Genotyping was conducted using a genome-wide single nucleotide polymorphism (SNP) method based on a Nextera-tagmented, reductively amplified DNA protocol (nextRAD [48]). NextRAD is a PCR-based, genotyping-by-sequencing protocol that can be applied to DNA of relatively low yield and quality [48].

In brief, genomic DNA was first fragmented with Nextera reagent (Illumina, Inc), which also ligates short adapter sequences to the ends of the fragments. The Nextera reaction was scaled for fragmenting 4 ng of genomic DNA, although 6 ng of genomic DNA was used for input to compensate for degraded DNA in the samples. Fragmented DNA was then amplified, with one of the primers matching the adapter and extending 8 nucleotides into the genomic DNA with the selective sequence GTATAAGG. Thus, only fragments starting with a sequence compatible with the selective sequence of the primer were efficiently amplified. The nextRAD libraries were sequenced on a HiSeq 2500 (University of Oregon, USA). The genotyping analysis used custom scripts (SNPsaurus, LLC, OR, USA) that created a de novo reference from abundant reads, and then mapped all reads to the reference with an alignment identity threshold of 95%. Read mapping and variant calling were done using samtools [49] and bcftools [50], respectively. Altogether, we genotyped 712 unique samples, from three populations, across 1887 SNP loci.

To characterize variation at *M*. *lini* infectivity loci we additionally PCR amplified and sequenced the ‘A’ genome alleles of the AvrP4 and AvrP123 infectivity genes using previously described methods [41]. Altogether 535 samples were used for Avr genotyping. (Kiandra, 356 samples: 1987–1992, 1996–2008; P1, 118 samples: 1987–1997; P2, 61 samples: 1987–1989, 1992–1997; Table 1). For the *AvrP4* gene we found 14 allelic variants. Two of these (Lm4 and Lm8) were present at high frequencies whereas other variants were relatively uncommon. At the *Avr123* gene we found two alleles (Lm1 and Lm2) that were present at high frequency, with an additional 29 singletons sampled. The singletons were treated as sequencing artefacts and removed from the analysis. We constructed multi-locus *AvrP4*/*AvrP123* haplotypes by combining the sequenced infectivity genes for each isolate. A total of five commonly occurring haplotypes were found.

### Analysis of genetic data

Prior to analysis, we removed uninformative loci (i.e. those at very low allelic frequency, representing 1–2 individuals) from the VCF file and excluded pathogen samples with >30% loci missing, leaving a total of 629 samples genotyped across 1567 polymorphic loci (Kiandra, 413 samples: 1987–2008; P1, 138 samples: 1987–1997, 2010; P2, 78 samples: 1987–1989, 1992–1997; Table 1).

### Clustering

Population subdivision into genetic clusters was performed using discriminant analysis of principal components (DAPC) [49]. DAPC was chosen because unlike some other clustering methods (e.g. STRUCTURE) it can be used for clonal or partially clonal populations [50]. The analysis was performed using the *find*.*clusters* function in *adegenet* package [49] in R software. For clustering, Prevosti’s distance, followed by principal component analysis was used. When calculating Prevosti’s distances and DAPC, the remaining missing data were ignored. Prevosti’s distance was used as it is a model free method and based on absolute differences between alleles [51, 52]. To retain the variation observed in the 1567 polymorphic loci, all 600 principal component axes were used to identify genetic clusters. Six clusters were ultimately identified as the most likely number based on the lowest BIC value and least subsequent increase or decrease in BIC values [49]. All 629 samples were retained in the DAPC analysis as the maximum number of clones of a MLG was low (1–13), and none of the clusters consisted of a single clone. To understand whether the two lineages of *M*. *lini* (AA vs. AB) that differ in their heterozygosity [42] were assigned to different clusters, we assessed heterozygosity among the obtained clusters. To understand how clusters are related, we used Prevosti’s distances to draw a neighbour-joining tree.

### Multi-locus genotypes

To estimate clonality in the populations, multi-locus genotypes (MLGs) were assigned using the *poppr* package [50] in R assuming a 5% genotyping error when inferring MLGs from a large highly variable clonal dataset. To assess the fraction of the observed variation arising from sequencing errors, three isolates were sequenced multiple times to estimate the genotyping error rate. The final 5% error rate used to filter the MLGs was determined based on the maximum observed error rate between these duplicates. A farthest neighbour algorithm and Prevosti’s distance matrix were then used to assign genotypes. While this enhances the likelihood of type I error, it significantly reduces the potential for type II error. Hence, we consider the approach is conservative in that overall it is more likely to result in an underestimate of the number of MLGs.

### Estimation of asexual vs sexual reproduction

To estimate the rate of potentially sexual reproduction and effective population size across years we performed analyses using *cloncase* R package [44] using the assigned MLGs for the whole population across years 1987–1992, and 1994–1997. We assumed eight cycles prior to the overwintering stage after first sampling, and eight cycles after overwintering stage prior to the second sampling. *Cloncase* is a program that implements algorithms to estimate the expected clonal and potentially sexual reproduction using MLG frequencies and the estimates of clonal cycles before and after the winter season. However, it does not use allele information obtained by SNP genotyping, and thus, does not produce any estimate of linkage between loci or similarity of MLGs.

We performed a pairwise homoplasy index (*Phi*) test for global recombination in *Splitstree* software [45] separately for populations and genetic clusters. The *Phi* test assesses the probability that recombination has occurred by comparing observed and permuted *phi* statistic (Φ), with significance set at 0.05. Observed linkage among loci was evaluated against permuted expected distributions using the index of association (I_A_) and *rD* [53–55]. I_A_ and *rD* were calculated using the *poppr* package [50] in R separately for the samples from each population and cluster. The significance of *I*_*A*_ values between loci was tested for each population with 999 permutations. Significant P values in Ia and *r*¯*d* therefore indicate linkage among loci.

### Spatio-temporal differentiation

To test whether isolates from different populations clustered separately, we performed principal component analysis (PCA) based on Prevosti’s distances of the SNP data. To understand how genetic variation was partitioned among populations, years, pathotypes and DAPC clusters, analyses of molecular variance (AMOVA) were performed using the *poppr* package [50] in R. Two datasets were generated for population genetic analyses: one that contained all isolates (n = 629) and one that contained only the isolates that were pathotyped using the differential set (n = 451). Two AMOVAs based on Prevosti’s distance [52] were conducted to investigate temporal dynamics. First, an analysis using the whole dataset was performed to partition variation among years at each study site (Kiandra, P1 and P2). Secondly, the dataset containing only pathotyped isolates was analysed to test for significant pathotypic variation among years at these sites. These analyses were performed both with and without clone correction. The significance of AMOVA results was tested using *randtest* in the R package *ade4* [56].

To understand how variation in infectivity genes was structured among populations, as well as within and between years, AMOVA was performed on infectivity gene alleles using the R software package *poppr*. Rare allelic variants (7.5% of the samples) that occurred only once in the dataset were excluded from this analysis.

### Population diversity

Overall population genetic richness and diversity was examined for the three populations as well as among years using the assigned MLGs. The expected number of genotypes was calculated using rarefaction in the R package *poppr*. To analyse diversity measured with SNPs (MLGs and clusters), infectivity gene haplotypes, and infectivity phenotypes within populations and across years, the Shannon index (*H*) was calculated [57–59] with the R package *poppr* [50].

### Statistical analyses

In order to understand whether measures of population diversity among years using different markers were correlated, we performed sets of Mantel tests for each population and for the whole data set. An ANOSIM analysis was performed using the R *vegan* package [43] to disentangle the variation caused by ecotype, population and year on the occurrence of genetic clusters, infectivity genotypes and phenotypes.

We performed another set of statistical analyses to understand: 1) the extent to which pathotype infectivity (i.e. the total proportion of differential hosts that were infected) and frequency in the pathogen population were correlated; 2) the relationship between the overall prevalence of pathotypes and the consistency of their presence across years; and 3) the relationship between infectivity as measured on the *L*. *marginale* differential set and infectivity measured on local or more distant host populations. We ran these analyses as Generalized Linear Mixed Models in SAS 9.4 PROC GLIMMIX (SAS Institute Inc. Cary, NC). We performed two analyses using data collected from the pathogen populations. To assess the relationship between pathotype infectivity and the prevalence of the pathotype each year we used infectivity as the response variable and prevalence as a covariate. Pathogen population was used as an explanatory class variable and year as a random variable. Infectivity was arcsine transformed to meet model requirements of normality. In the second model, in order to examine the extent to which strain prevalence in a population correlated with length of time (years) it was resident, pathogen population was analysed as an explanatory class variable and mean prevalence of each pathotype was used as a covariate. A Poisson distribution of error was assumed for years of pathogen prevalence. To test whether infectivity measured on differential hosts correlated with infectivity on sympatric and allopatric hosts, data collected from six populations, G1, G3, SH1, SH2, WHP1 and WHP2 was used. The mean infectivity of pathogen strains as measured on differential hosts was regressed against its mean infectivity on sympatric hosts and mean infectivity on allopatric hosts. To increase normality, infectivity data were arcsine transformed.

## Discussion

Pathogen population genetic structure is greatly influenced by evolutionary processes and epidemiology [1, 2], however we are only beginning to understand how ecological and environmental factors interact to determine evolution in wild pathogen populations. This is particularly the case for plant pathogens given that the majority of studies on plant pathogen populations have focused on interactions in agricultural [60, 61] or forestry [12, 62] contexts. Theoretical predictions [63, 64] and empirical studies in agricultural systems [16] have shown epidemiological dynamics and host mediated selection to be crucial in shaping pathogen population structure. However, we lack long-term studies that focus on how these forces drive genetic change in wild systems. Genotyping and statistical analysis of three natural *M*. *lini* pathogen populations across 16 annual epidemics identified the presence of six clusters of genetically related individuals. Using this framework, we were able to evaluate the relative importance of spatial and temporal aspects in maintaining pathogen variation. We found that genetic variation was maintained through time via among-population differentiation, and that pathogen populations are genetically and phenotypically dynamic (see also [17]). We also found a temporal shift in the prevalence of one major cluster in two populations, consistent with directional selection. Against expectations, our results support the concept that both clonality and recombination (potentially via somatic hybridization) play a role in the population structure of *M*. *lini*.

### Host ecotype drives selection but pathotype may originate from multiple genetic sources

Using the clusters identified through analysis of extensive genotype and phenotype data, we were able to evaluate the relative importance of spatial and temporal scale in maintaining variation in pathogen populations. While pathogen populations were genetically and phenotypically dynamic through time, we found that strong spatial structuring among populations was the predominant factor maintaining the different genetic clusters within the metapopulation over time in the Kiandra and P1 populations. Despite their close physical proximity, strong fluctuations in population size and severe annual bottlenecks, these populations are clearly differentiated from each other in terms of genotypic composition (i.e clusters) and diversity. Similarly, Avr haplotypes and pathotype composition were strongly differentiated among populations but showed only small temporal changes. These results are consistent with data on host genetic structure, whereby populations of genetically discrete ecotypes (hill and bog) are maintained over time [36].

Both SNP and infectivity gene diversity were higher in the Kiandra and P1 populations than in P2. This may reflect in part the higher resistance and infectivity diversity observed previously in Kiandra [36]. Genetic Cluster 3 had comparatively low heterozygosity (suggesting an AA lineage origin [42]) and was present at low frequencies in both Kiandra and P1. The sampling of common clusters among populations suggested the potential for significant between-population migration as well as longer distance dispersal from the more distant plains region, as would be expected in a metapopulation situation.

Pathotyping using the *L*. *marginale* differential set showed that *L*. *marginale* populations hosted a diversity of pathotypes in each year, the majority of which went extinct during the following overwintering period. Only a minority of pathotypes persisted over the entire study period and were present in all three populations. This result is consistent with the high MLG diversity revealed by SNP genotyping. While the among-year turnover of pathotypes was high, population pathotype structure was strongly influenced by ecotype (bog vs. hill). Pathotype A generally dominated in Kiandra, while four different pathotypes (including A) formed a major component of the other populations. This is consistent with previous studies on this system [36, 65]. However, the patterns observed for pathotype and genetic diversity were partially contrasting, such that pathotype analysis revealed a small number of dominant pathotypes, while genotyping revealed high diversity and low annual turnover within populations.

### Temporal changes in population structure

As for many plant pathogens, annual variation in the intensity of epidemics and seasonal bottlenecks are expected to strongly shape the genetic dynamics and evolutionary potential of *M*. *lini* populations [1]. At the beginning of winter, *L*. *marginale* plants die back to rootstocks, meaning that the foliar tissue required to sustain this biotrophic pathogen is largely absent. The infection cycle begins in spring from infections thought to have survived on scarce foliar tissue or from spores migrating from other populations. Our results indicate that both life history and environmental conditions strongly influence pathogen genetic dynamics and population structure. For example, in the Kiandra population in 1994 and 1995, this bottleneck was followed by a poor epidemic, which resulted in only a small number of isolates from a single cluster being sampled. Thus, genetic diversity can be greatly limited by demographic processes in some years. Conversely, despite the sample being dominated by strains resulting from asexual production in most years, assigned genotypes (MLGs) were largely ephemeral and unlikely to persist between growing seasons, with over 80% of genotypes sampled only once. Overall, while we cannot rule out cryptic local infections as a source of epidemics, our results suggest that the broader metapopulation is a crucial genetic reservoir, in that high levels of genotypic diversity are maintained at larger spatial scales, and migration from other populations appears to be important in re-establishing local pathogen populations at the start of epidemics.

Our findings also support a role for selection in driving temporal change in population genetic and phenotypic structure. For example, a clear genetic turnover event was observed in Kiandra in 2004, when the composition of the entire sampled population shifted to be dominated by a single genetic cluster (Cluster 2 isolates; Fig 2C). Prior to 2004, Cluster 1 isolates were consistently sampled and often represented the dominant component of the Kiandra population while isolates belonging to Cluster 2 were mostly rare and only present in combination with Cluster 1 isolates. Subsequent to the 2004 genetic shift, isolates grouping within other clusters dispersed into the population, whereas Cluster 1 isolates disappeared. The change in the dominating cluster frequency is consistent with directional selection and a selective sweep operating in the population. However, we cannot rule out the possibility that other random processes in metapopulation level e.g. drift or migration could have been at least partly responsible for the observed fluctuations in cluster frequencies.

### Annual epidemics are polymorphic and partly clonal

We found significant LD within most years, populations and clusters, which is consistent with non-random mating and potential clonal reproduction [66]. However, the high levels of genotypic diversity found within populations was an unexpected result. *M*. *lini* was thought to be almost exclusively asexual in this region [10, 46], and low levels of genetic and genotypic polymorphism have been reported in previous studies (albeit with a far lower number of markers [41]). Indeed, in contrast to theoretical expectations for asexual pathogens on epidemic growth being dominated by few genotypes [2, 66], and our own expectations based on observations of limited pathotype diversity within epidemic development [67], our observations of high MLG diversity within years suggests that clonal spread from a small number of source genotypes is limited. Higher than expected levels of within-population diversity are consistent with some form of genetic exchange (e.g. sexual reproduction) among individuals [68, 69], and results from *Phi* tests indicate that genetic recombination is common to all clusters and populations. In addition, Avr alleles and haplotype combinations were often shared between clusters. This further suggests that there is likely some genetic exchange among groups. Thus, while clonal spread may occur, it does not appear to be the dominant driving force in epidemic development or the generation of population genetic structure.

Several recent studies of clonal or partially clonal pathogens have reported similarly low levels of clonality. A recent study of the closely related pathogen *Melampsora larici-populina* showed clonal spread only at very fine spatial scales within leaf and twig [11]. Similarly, clonality was observed mainly at the smallest spatial scales in *Sclerotinia sclerotinium* [70], while high diversity within populations and variable spread of single pathogen lineages during epidemics was reported for *Plasmopora viticola* [71]. These studies emphasize the need to investigate clonal spread of pathogens at multiple spatial scales starting from within host scale. Our sampling design aimed to representatively sample the whole population leaving fine scale dynamics unexplored. While we acknowledge that the effective pathogen population size was larger than what was possible to sample, the sampling effort per year was proportional to the pathogen population in a given year. Thus, we sampled at a sufficient depth to capture a representative amount of diversity in the populations.

Somatic hybridization is one mechanism by which genetic exchange among asexual fungi can be facilitated [72, 73], and involves the fusion of hyphae (i.e. anastomosis), followed by genetic exchange via processes such as heterokaryosis, nuclear fusion, recombination, and reassortment of chromosomes. The frequency at which such events occur under natural conditions is unresolved, but somatic hybridization has been hypothesized as a mechanism for generating unexpectedly high levels of genetic and pathogenic variation in populations of several asexual rust pathogens of crop plants (e.g. *Phakopsora pachyrhizi*)[73–75], *Puccinia striiformis* [76] and *P*. *graminis* f. sp. *tritici* [73]). In our study, the estimated (para)sexual reproduction rate ranged from 0 to 99.8 suggesting that there may be specific factors that trigger parasexual reproduction (e.g. coinfection; environmental conditions) [77], or alternatively, very strong selection on clones or recombinants. Another possibility is that, in presence of migration from the wider metapopulation, the rate at which new genetic variants are generated is overestimated as the *Cloncase* analysis does not account for migration from other populations. Migration may also bias the *Cloncase* estimated *Ne*.

One caveat is that although local population structure appeared to be highly dynamic in space and time, we assigned MLGs by estimating the error rate based on a set of duplicate samples and cannot exclude the possibility that some of the MLG dynamics observed are due to the method of assigning MLGs. The same MLGs were recaptured over successive years which is a pattern that could arise either because a given MLG is persisting stably in a population [69] or because we underestimated diversity by assuming a 5% error rate.

### Pathotypes have evolved multiple times

We found no direct correlation between pathotypes and genotypes although pathotypes explained 25% of the variation in the AMOVA. Varying correlations between different marker types have been reported in other studies. Kohn [78] investigated correlations between pathotypes and genotypes in wild populations of *S*. *sclerotinium* and found no correlation between mycelial compatibility groups and genotypes even though in agricultural populations such associations occurred [78]. Similar patterns have been previously reported in cereal rusts [79, 80], other fungal pathogens [81] and nematodes [82]. In contrast, studies of some systems have found a clear pathotype-genotype correlation (one pathotype represents one genotype and vice versa; e.g. *Verticillium dahlia* [83]). Other studies have shown that different pathotypes can arise within the same clonal lineage [84, 85] suggesting rapid evolution of pathogenicity. While sexual recombination is often hard to observe in wild, its occurrence may have been underestimated in past studies. However, in *M*. *lini* we observed genetic diversity within pathotypes similar to that found in *M*. *larici-populina*, where the same pathotype could be represented by several genotypes [40]. In other rusts, such as *P*. *graminis*, high within population diversity has been reported on both wheat landraces [79] and susceptible cultivars [80].

In modern agricultural systems, selection often leads to the dominance of infectious pathotypes that are genetically uniform [86, 87]. Our results showed that a small number of pathotypes dominated populations and that in the Kiandra *L*. *marginale* population in which high levels of resistance occur [88], one locally universally infectious pathotype A [38] prevailed throughout the study. However, genotyping revealed that isolates of pathotype A were neither genetically identical nor clearly phylogenetically clustered. Prediction of pathogen evolutionary trajectories as well as the application of such knowledge to the development of effective disease management strategies in cropping systems, requires integration of information on both phenotypic and genotypic diversity.

## Conclusions

This is one of the first studies to explicitly link long-term epidemiological, genetic and pathotypic dynamics in the wild. Analyses of pathotypes clearly showed that selection at the level of host ecotype (bog vs. hill) leads to the maintenance of specialist pathogens within populations over time. Interestingly, the diversity obtained by SNP genotyping and the lack of direct links between MLGs, pathotypes and infectivity gene variation suggests that polymorphism arises from multiple origins rather than diversification of one or few clonal lineages. High levels of among-population migration are also supported by evidence for multiple colonisations by AA lineage isolates. Together with varying frequency of clonal patterns, these findings give new insights into the evolutionary potential of pathogen populations in the wild and emphasize the need for long-term studies as well as directions for future avenues of research. Thus, to fully understand the contribution of clonal dynamics to natural host-pathogen interactions, studies spanning multiple hierarchical levels are needed. We propose that characterising the sources of such hidden genetic diversity and its impact on disease evolution and spread is of crucial importance to engineering strategies to deploy durable resistance and mitigate the harmful effects of epidemics in managed systems.

## Supporting information

S1 Fig Neighbour–joining tree of *Melampsora lini* isolates collected from the populations Kiandra, P1, and P2 across years 1987–2010. The colours indicate genetic clusters purple = 1, blue = 2, orange = 3, brown = 4, red = 5, and green = 6.
(TIF)

S2 Fig The expected number of *Melampsora lini* multilocus genotypes in populations Kiandra (K), P1 and P2 analysed with rarefaction. (TIF)

S1 Table Results of hierarchical analysis of molecular variance (AMOVA) obtained by genome wide SNP genotyping for isolates of *Melampsora lini* originating from three populations (Kiandra, P1 and P2) on clone corrected data over years 1987–2008. Results are presented from the whole dataset (N = 629) and the dataset on the *Melampsora lini* isolates that were pathotyped (N = 451), and infectivity gene genotyped (N = 539).
(DOCX)

S2 Table Results on Analysis of linkage disequilibrium and *Phi* test for recombination in SNP genotyped isolates of *Melampsora lini* six genetic clusters. Linkage disequilibrium as index of association (*Ia*) and *rD*. Significant *Ia* and *rD* values indicate linkage among loci. *Phi* test results as mean and observed linkage. Significant *P* values in *Phi* test indicate recombination.
(DOCX)

S3 Table Results on *Phi* test for recombination in SNP genotyped isolates of *Melampsora lini in* populations Kiandra, P1, and P2 across years. *Phi* test results on mean and observed linkage, and *P* value for probability of recombination.
(DOCX)

S4 Table Analysis of linkage disequilibrium in SNP genotyped isolates of *Melampsora lini* in populations Kiandra, P1, and P2 across years. Linkage disequilibrium as index of association (*Ia*), *rD*, and *P* values indicating probability of significant levels of LD.
(DOCX)
