# Supplementary figures and images for: Genetic analysis reveals long-standing population differentiation and high diversity in the rust pathogen Melampsora lini
Source: PLoS Pathog. 2020 Aug 18;16(8):e1008731. doi: 10.1371/journal.ppat.1008731 (PMC7454959; doi:10.1371/journal.ppat.1008731)

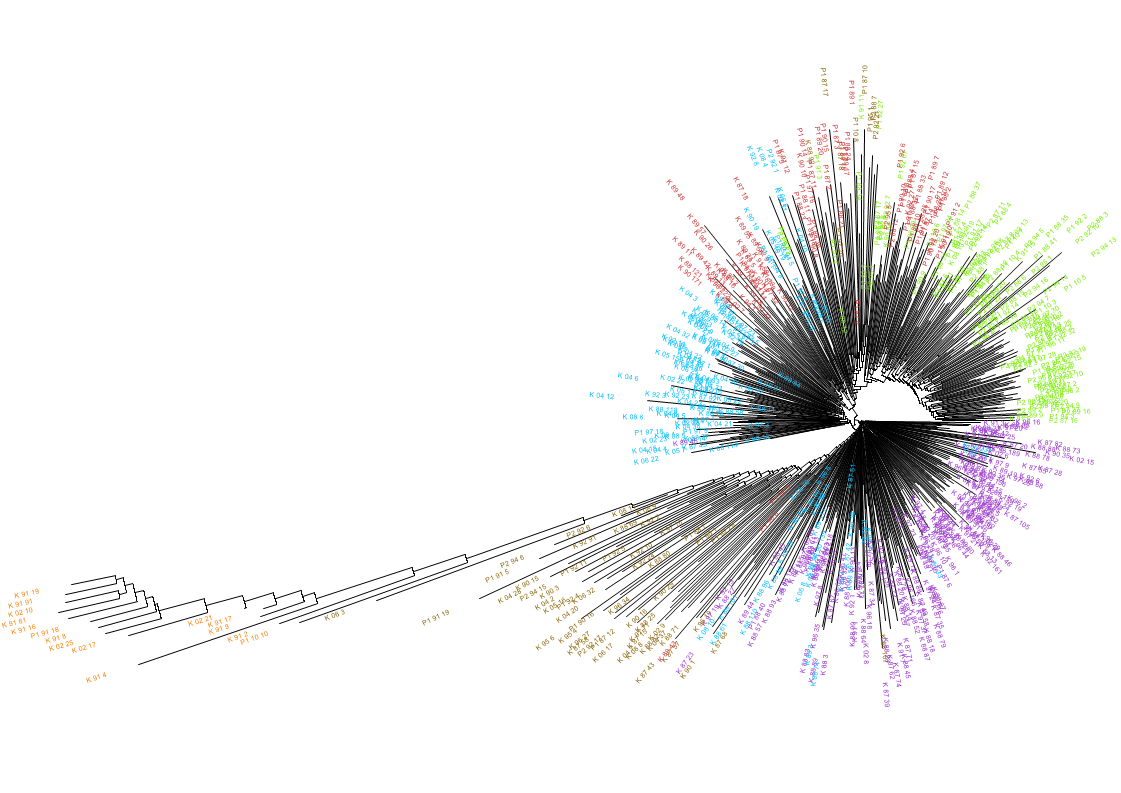

Supplement: S1 Fig — The colours indicate genetic clusters purple = 1, blue = 2, orange = 3, brown = 4, red = 5, and green = 6. (TIF) [file ppat.1008731.s001.tif]

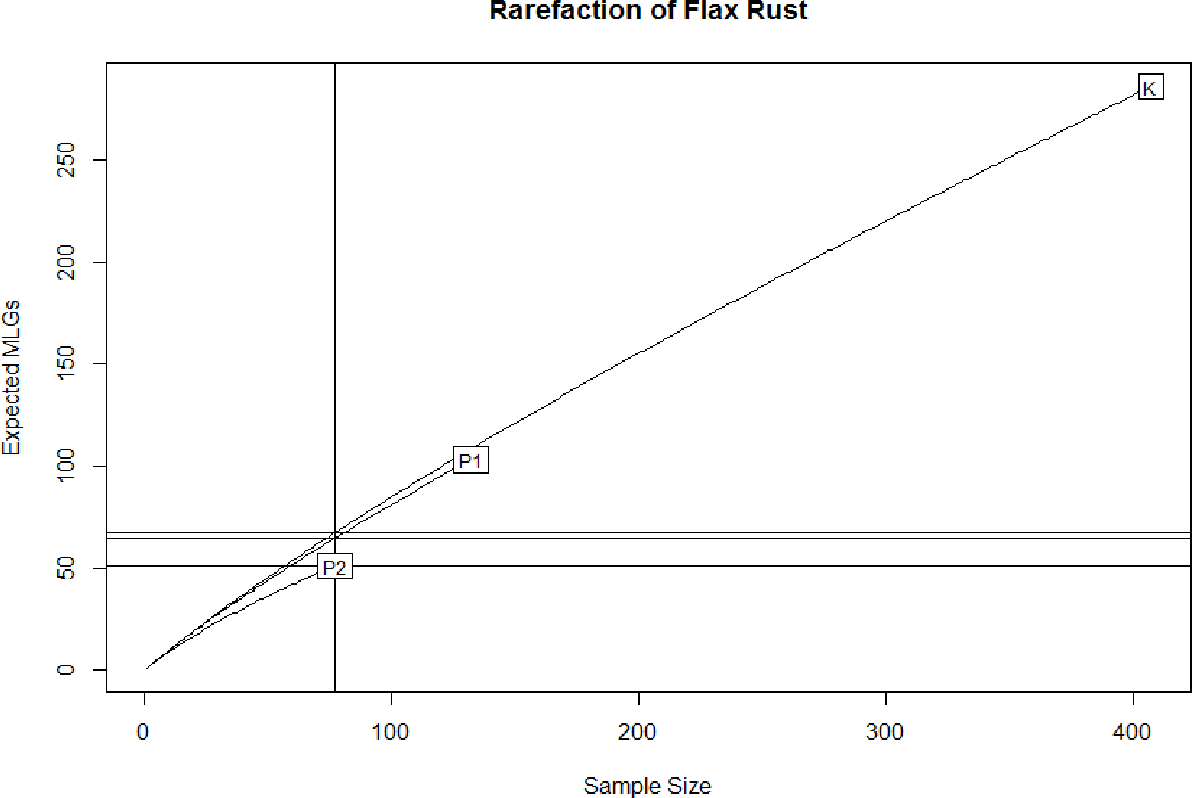

Supplement: S2 Fig — (TIF) [file ppat.1008731.s002.tif]
