# Supplementary material for: Genetic analysis reveals long-standing population differentiation and high diversity in the rust pathogen Melampsora lini
Source: PLoS Pathog. 2020 Aug 18;16(8):e1008731. doi: 10.1371/journal.ppat.1008731 (PMC7454959; doi:10.1371/journal.ppat.1008731)
Supplement: S1 Table — Results are presented from the whole dataset (N = 629) and the dataset on the Melampsora lini isolates that were pathotyped (N = 451), and infectivity gene genotyped (N = 539). (DOCX) [file ppat.1008731.s003.docx]

**S1 Table.**

| **SNP genotyping** | **AMOVA with clonecorrection** | |  |  |  |
| --- | --- | --- | --- | --- | --- |
|  | ***Df*** | ***Sum of Squares*** | ***Mean Squares*** | ***% Variance*** | ***P*** |
| **Between populations** | 2 | 0.077 | 0.0387 | 3.2 | 0.001 |
| **Between years within populations** | 30 | 0.29 | 0.009 | 3.2 | 0.002 |
| **Within populations within pathotypes within years within year** | 521 | 3.23 | 0.0062 | 93.6 | 0.001 |
| **Total** | 553 | 3.59 | 0.0065 | 100 |  |
| **Between populations** | 2 | 0.08 | 0.042 | -5.5 | 0.243 |
| **Within populations between pathotypes** | 73 | 1.03 | 0.014 | 30.2 | 0.001 |
| **Within populations within pathotypes between years** | 62 | 0.33 | 0.005 | -0.42 | 0.532 |
| **Within populations within pathotypes within years within year** | 318 | 1.74 | 0.005 | 75.8 | 0.001 |
| **Total** | 455 | 3.2 | 0.007 | 100 |  |
