# Supplementary material for: Genetic analysis reveals long-standing population differentiation and high diversity in the rust pathogen Melampsora lini
Source: PLoS Pathog. 2020 Aug 18;16(8):e1008731. doi: 10.1371/journal.ppat.1008731 (PMC7454959; doi:10.1371/journal.ppat.1008731)
Supplement: S2 Table — Linkage disequilibrium as index of association (Ia) and rD. Significant Ia and rD values indicate linkage among loci. Phi test results as mean and observed linkage. Significant P values in Phi test indicate recombination. (DOCX) [file ppat.1008731.s004.docx]

**S2 Table.**

|  |  | **All** |  |  |  | **Kiandra** | |  |  | **P1** |  |  |  | **P2** |  |  |  |
| --- | --- | --- | --- | --- | --- | --- | --- | --- | --- | --- | --- | --- | --- | --- | --- | --- | --- |
| **Index of association** | **Cluster** | ***Ia*** | ***P*** | ***rD*** | ***P rD*** | ***Ia*** | ***P*** | ***rD*** | ***P rD*** | ***Ia*** | ***P*** | ***rD*** | ***P rD*** | ***Ia*** | ***P*** | ***rD*** | ***P rD*** |
|  | **1** | 72.27 | 0.001 | 0.05 | 0.001 | 93.14 | 0.001 | 0.066 | 0.001 | 24.82 | 0.001 | 0.019 | 0.001 | 11.58 | 0.001 | 0.177 | 0.001 |
|  | **2** | 56.18 | 0.001 | 0.03 | 0.001 | 102.2 | 0.001 | 0.072 | 0.001 | 22.06 | 0.001 | 0.018 | 0.001 | 32.97 | 0.001 | 0.026 | 0.001 |
|  | **3** | 299.44 | 0.001 | 0.21 | 0.001 | 219.3 | 0.001 | 0.234 | 0.001 | 3.553 | 0.004 | 0.08 | 0.004 | - | - | - | - |
|  | **4** | 80.38 | 0.001 | 0.05 | 0.001 | 84.34 | 0.001 | 0.058 | 0.001 | 118 | 0.001 | 0.096 | 0.001 | 24.07 | 0.001 | 0.034 | 0.001 |
|  | **5** | 97.52 | 0.001 | 0.07 | 0.001 | 91.31 | 0.001 | 0.069 | 0.001 | 41.17 | 0.001 | 0.034 | 0.001 | 82.58 | 0.001 | 0.015 | 0.001 |
|  | **6** | 61.74. | 0.001 | 0.04 | 0.001 | 102.2 | 0.001 | 0.072 | 0.001 | 22.06 | 0.001 | 0.019 | 0.001 | 32.97 | 0.001 | 0.026 | 0.001 |
|  | **All** | 85.04 | 0.001 | 0.058 | 0.001 | 52.47 | 0.001 | 0.036 | 0.001 | 62.57 | 0.001 | 0.042 | 0.001 | 27.5 | 0.001 | 0.021 | 0.001 |
| **Phi test** | | **Mean** | **Variance** | **Obs.** | ***P*** | **Mean** | **Variance** | **Obs.** | ***P*** | **Mean** | **Variance** | **Obs.** | ***P*** | **Mean** | **Variance** | **Obs.** | ***P*** |
|  | **1** | 0.8121 | 0.0000 | 0.808 | 0.0001 | 0.745 | 0.0000 | 0.743 | 0.067 | 0.436 | 0.0000 | 0.43 | 0.002 | 0.467 | 0.0000 | 0.4563 | 0.0363 |
|  | **2** | 0.7764 | 0.0000 | 0.773 | 0.006 | 0.665 | 0.0000 | 0.662 | 0.048 | 0.55 | 0.0000 | 0.545 | 0.001 | 0.444 | 0.0000 | 0.4123 | 0.0000 |
|  | **3** | 0.3643 | 0.0000 | 0.361 | 0.013 | 0.273 | 0.0000 | 0.27 | 0.063 | 0.511 | 0.0001 | 0.468 | 0.0000 | - | - | - | - |
|  | **4** | 0.6841 | 0.0000 | 0.679 | 0.0001 | 0.603 | 0.0000 | 0.606 | 0.983 | 0.403 | 0.0000 | 0.397 | 0.02 | 0.517 | 0.0000 | 0.4983 | 0.0018 |
|  | **5** | 0.6424 | 0.0000 | 0.636 | 0.0001 | 0.477 | 0.0000 | 0.47 | 0.0001 | 0.517 | 0.0000 | 0.512 | 0.011 | 0.528 | 0.0000 | 0.5065 | 0.0001 |
|  | **6** | 0.76 | 0.0000 | 0.754 | 0.049 | 0.606 | 0.0000 | 0.607 | 0.613 | 0.487 | 0.0000 | 0.486 | 0.418 | 0.433 | 0.0000 | 0.4251 | 0.0001 |
|  | **All** | 0.9721 | 0.0000 | 0.969 | 0.0001 | 0.947 | 0.0000 | 0.942 | 0.0001 | 0.776 | 0.0000 | 0.773 | 0.007 | 0.486 | 0.0000 | 0.4821 | 0.0071 |
