# Supplementary material for: Genetic analysis reveals long-standing population differentiation and high diversity in the rust pathogen Melampsora lini
Source: PLoS Pathog. 2020 Aug 18;16(8):e1008731. doi: 10.1371/journal.ppat.1008731 (PMC7454959; doi:10.1371/journal.ppat.1008731)
Supplement: S3 Table — Phi test results on mean and observed linkage, and P value for probability of recombination. (DOCX) [file ppat.1008731.s005.docx]

**S3 Table.**

|  | **Kiandra** | |  |  | **P1** |  |  |  | **P2** |  |  |  |
| --- | --- | --- | --- | --- | --- | --- | --- | --- | --- | --- | --- | --- |
| **Year** | Mean | Variance | Obs. | *P* | Mean | Variance | Obs. | *P* | Mean | Variance | Obs. | *P* |
| 1987 | 0.54 | 0.0000 | 0.53 | 0.0082 | 0.51 | 0.00 | 0.50 | 0.2931 | 0.51 | 0.00 | 0.52 | 0.9794 |
| 1988 | 0.56 | 0.0000 | 0.56 | 0.0650 | 0.50 | 0.00 | 0.49 | 0.0005 | 0.48 | 0.00 | 0.47 | 0.0001 |
| 1989 | 0.55 | 0.0000 | 0.55 | 0.0009 | 0.50 | 0.00 | 0.48 | > 0.0001 | 0.61 | 0.00 | 0.62 | 0.5249 |
| 1990 | 0.51 | 0.0000 | 0.50 | 0.0107 | 0.41 | 0.00 | 0.40 | 0.0041 | - | - | - | - |
| 1991 | 0.53 | 0.0000 | 0.52 | 0.0000 | 0.42 | 0.00 | 0.41 | > 0.0001 | - | - | - | - |
| 1992 | 0.39 | 0.0000 | 0.39 | 0.3307 | 0.40 | 0.00 | 0.39 | > 0.0001 | 0.41 | 0.00 | 0.40 | > 0.0001 |
| 1994 | - | - | - | - | 0.52 | 0.00 | 0.48 | > 0.0001 | 0.42 | 0.00 | 0.39 | > 0.0001 |
| 1995 | - | - | - | - | - | - | - | - | 0.48 | 0.00 | 0.46 | 0.0062 |
| 1996 | 0.52 | 0.0000 | 0.52 | 0.5599 | 0.46 | 0.00 | 0.43 | 0.0040 | 0.65 | 0.00 | 0.56 | 0.0076 |
| 1997 | 0.50 | 0.0000 | 0.49 | 0.3112 | 0.49 | 0.00 | 0.48 | 0.0055 | - | - | - | - |
| 2002 | 0.37 | 0.0000 | 0.36 | 0.0137 | - | - | - | - | - | - | - | - |
| 2004 | 0.51 | 0.0000 | 0.51 | 0.2617 | - | - | - | - | - | - | - | - |
| 2005 | 0.51 | 0.0000 | 0.50 | 0.0020 | - | - | - | - | - | - | - | - |
| 2006 | 0.52 | 0.0000 | 0.50 | 0.0000 | - | - | - | - | - | - | - | - |
| 2008 | 0.36 | 0.0000 | 0.35 | 0.0018 | - | - | - | - | - | - | - | - |
| 2010 | - | - | - | - | 0.51 | 0.00 | 0.49 | 0.0000 | - | - | - | - |
