# Supplementary material for: Genetic analysis reveals long-standing population differentiation and high diversity in the rust pathogen Melampsora lini
Source: PLoS Pathog. 2020 Aug 18;16(8):e1008731. doi: 10.1371/journal.ppat.1008731 (PMC7454959; doi:10.1371/journal.ppat.1008731)
Supplement: S4 Table — Linkage disequilibrium as index of association (Ia), rD, and P values indicating probability of significant levels of LD. (DOCX) [file ppat.1008731.s006.docx]

**S4 Table.**

|  | **Kiandra** | |  |  | **P1** |  |  |  | **P2** |  |  |  | **All** |  |  |  |
| --- | --- | --- | --- | --- | --- | --- | --- | --- | --- | --- | --- | --- | --- | --- | --- | --- |
| **Year** | *Ia* | *P* | *rD* | *P rD* | *Ia* | *P* | *rD* | *P rD* | *Ia* | *P* | *rD* | *P rD* | *Ia* | *P* | *rD* | *P rD* |
| 1987 | 28.4 | 0.001 | 0.022 | 0.001 | 27.17 | 0.017 | 0.024 | 0.019 | 9.03 | 0.001 | 0.016 | 0.001 | 28.34 | 0.001 | 0.02 | 0.001 |
| 1988 | 18.31 | 0.001 | 0.017 | 0.001 | 8.784 | 1.000 | 0.008 | 1.000 | 19.88 | 0.001 | 0.023 | 0.001 | 14.76 | 0.001 | 0.01 | 0.001 |
| 1989 | 18.32 | 0.001 | 0.016 | 0.001 | 17.66 | 0.001 | 0.021 | 0.001 | 4.31 | 0.909 | 0.023 | 0.641 | 20.12 | 0.001 | 0.017 | 0.001 |
| 1990 | 39.13 | 0.001 | 0.033 | 0.001 | 42.43 | 0.001 | 0.047 | 0.001 | - | - | - | - | 38.32 | 0.001 | 0.031 | 0.001 |
| 1991 | 219.1 | 0.001 | 0.154 | 0.001 | 199.6 | 0.001 | 0.147 | 0.001 | - | - | - | - | 174 | 0.001 | 0.12 | 0.001 |
| 1992 | 19.9 | 1.000 | 0.016 | 1.000 | 18.89 | 1.000 | 0.016 | 1.000 | - | - | - | - | 21 | 1 | 0.015 | 1 |
| 1994 | - | - | - | - | 5.684 | 0.001 | 0.014 | 0.001 | 31.28 | 0.001 | 0.029 | 0.001 | 39.57 | 0.001 | 0.037 | 0.001 |
| 1995 | - | - | - | - | 32.98 | 0.001 | 0.075 | 0.001 | 43.54 | 0.001 | 0.045 | 0.001 | 51.9 | 0.001 | 0.049 | 0.001 |
| 1996 | 21.1 | 0.001 | 0.019 | 0.001 | 4.73 | 1.000 | 0.011 | 1.000 | 12.42 | 0.001 | 0.025 | 0.001 | 19.28 | 0.001 | 0.017 | 0.001 |
| 1997 | 12.29 | 0.193 | 0.132 | 0.132 | 10.51 | 0.964 | 0.013 | 0.967 | 8.599 | 0.054 | 0.049 | 0.004 | 10.8 | 0.986 | 0.01 | 1 |
| 2002 | 224.8 | 0.001 | 0.165 | 0.001 | - | - | - | - | - | - | - | - | 224.8 | 0.001 | 0.165 | 0.001 |
| 2004 | 32.53 | 0.001 | 0.025 | 0.001 | - | - | - | - | - | - | - | - | 32.52 | 0.001 | 0.025 | 0.001 |
| 2005 | 21.7 | 0.001 | 0.023 | 0.001 | - | - | - | - | - | - | - | - | 21.7 | 0.001 | 0.024 | 0.001 |
| 2006 | 72.81 | 0.001 | 0.064 | 0.001 | - | - | - | - | - | - | - | - | 72.82 | 0.001 | 0.064 | 0.001 |
| 2008 | 27.89 | 1.000 | 0.028 | 1.000 | - | - | - | - | - | - | - | - | 27.89 | 1 | 0.028 | 1 |
| 2010 | - | - | - | - | 160.4 | 0.001 | 0.151 | 0.001 | - | - | - | - | 160.4 | 0.001 | 0.151 | 0.001 |
| **All** | **52.47** | **0.001** | **0.036** | **0.001** | **62.57** | **0.001** | **0.042** | **0.001** | **27.5** | **0.001** | **0.021** | **0.001** | **85.04** | **0.001** | **0.058** | **0.001** |
